# A Low Noise Cascode Amplifier

**DOI:** 10.6028/jres.092.039

**Published:** 1987-12-01

**Authors:** Steven R. Jefferts, F. L. Walls

**Affiliations:** Joint Institute for Laboratory Astrophysics, University of Colorado and National Bureau of Standards Boulder, CO 80309; National Bureau of Standards Boulder, CO 80303

**Keywords:** cascode amplifier, low bias current amplifier, low noise FET amplifier, noise analysis, noise current, noise voltage

## Abstract

We describe the design, schematics, and performance of a very low noise FET cascode input amplifier. This amplifier has noise performance of less than $1.2\;\text{nV}/\sqrt{\text{Hz}}$ and 0.25 $0.25\;\text{fA}/\sqrt{\text{Hz}}$ over the 500 Hz to 50 kHz frequency range. The amplifier is presently being used in conjunction with a Penning ion trap but is applicable to a wide variety of uses requiring low noise gain in the 1 Hz to 30 MHz frequency range.

## Introduction

A low noise amplifier has been designed using a 2SK117 N channel J-FET as the input device in a cascode [1] configuration. Noise measurements on this amplifier yield a low frequency noise current of $0.25\;\text{fA}/\sqrt{\text{Hz}}$ and a voltage noise of less than $1.2\;\text{nV}/\sqrt{\text{Hz}}$ in the 500 Hz to 50 kHz region. Bloyet *et al.* [2] suggest a figure of merit of the product of the noise voltage and current as being appropriate for amplifiers of this type. This amplifier has a figure of merit of ~3×10^−25^ W/Hz, which is almost two orders of magnitude smaller than other amplifiers reported elsewhere. [2]

The amplifier described here is presently being used in conjunction with a Penning trap to detect small image currents (~0.01 pA) induced by ion motion in the trap. [3] This amplifier also appears to be well suited for use in noise thermometry experiments. [4]

This paper discusses some general design criteria for cascode amplifiers and draws some conclusions concerning the optimum choice of FETs for such amplifiers. A particular design having the noise performance described above is presented and analyzed. Variations of the design which either have much larger bandwidth, 30 MHz, or draw extremely low input bias current, less than 0.01 pA, are briefly discussed.

## Equivalent Circuit for Noise Analysis

**Figure 1:**
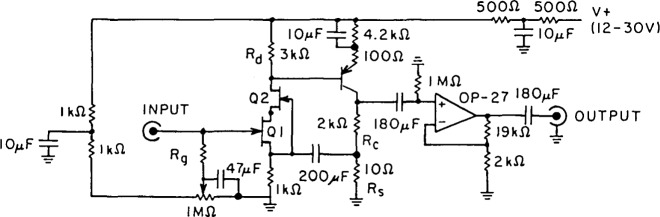
Schematic diagram of the low noise preamplifier. NOTE: 1) All resistors 1% metal film. 2) All capacitors are tantalum. 3) V+ must be well filtered. 4) The OP-27 power supply leads should be bypassed with 10 Ω and 0.1 *μ*F close to the OP-AMP.

The schematic of the amplifier is shown in figure 1. The biasing scheme used for Q2, the common base portion of the cascode, is attractive for its simplicity and inherent low noise. However, to work properly it requires that *I*dss [5] of Q2 be larger than *I*dss of Q1.

**Figure 2:**
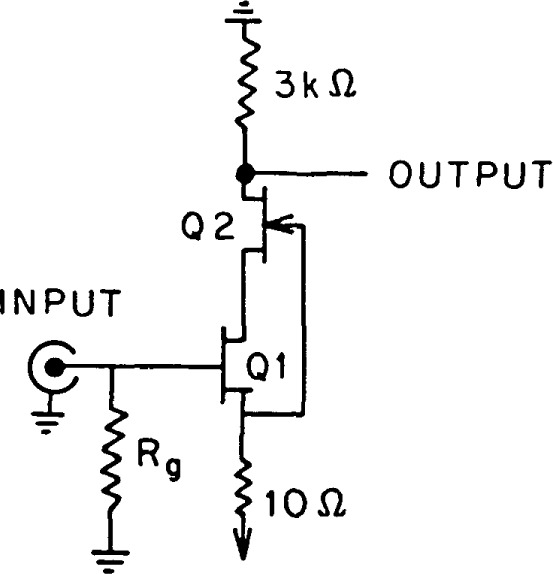
Signal frequency equivalent circuit of the cascode input stage.

**Figure 3:**
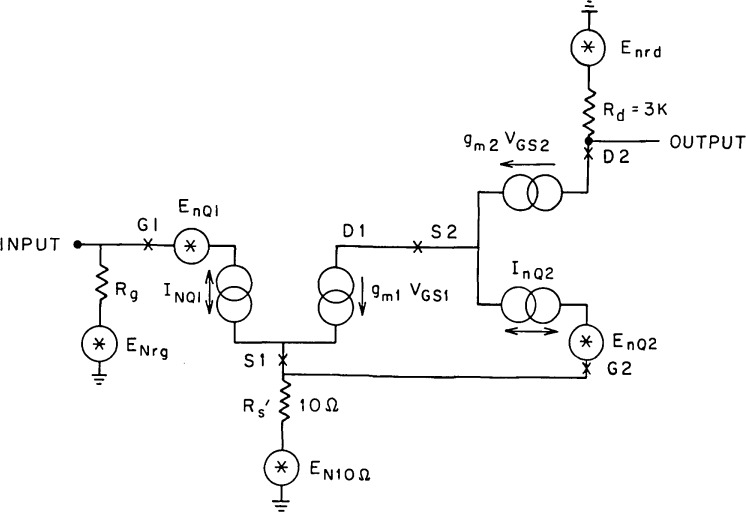
The noise equivalent circuit of the cascode input state.

The gain of the cascode input stage is large, about 50. Hence, the noise in this stage is the dominant noise mechanism in the amplifier, and we will therefore confine our analysis to the cascode input stage and the associated biasing circuitry. The signal frequency equivalent circuit of the input stage is illustrated in figure 2. From figure 2 we can proceed to draw the noise equivalent circuit as shown in figure 3. Using this model, we can write the equivalent input noise, *E*_ni_, as [6]

$$\begin{array}{r} E_{\text{ni}}^{2}=E_{nR_{g}}^{2}+E_{\text{nQ}1}^{2}+I_{\text{nQ}1}^{2}Z_{g}^{2}+{\left(\frac{1}{K_{Q1}}\right)}^{2}\\ \left[E_{\text{nQ}2}^{2}+I_{\pi Q2}^{2}R_{s1}^{2}\right]+{\left(\frac{1}{K_{t}}\right)}^{2}E_{nR_{d}}^{2} \end{array}$$ (1)

where *K*_t_ = gm1*R*_d_ and *K*_Q1_ = −gm1/gm2 is the gain of the common source component of the cascode. Z_g_ is the impedance presented to the gate of Q1 formed by the parallel combination of *C*_g_, the gate capacitance, and *R*_g_, the gate bias resistor. The choice of Q2 is governed by a tradeoff between bootstrapping *C*_gd_ of Q1 for lowest input capacitance and gain in Q1 suppressing voltage noise in Q2 relative to Q1. This suggests that the choice of identical FETs for Q1 and Q2 may not be optimum. For this amplifier, we chose Q2 to be a 2N4416, yielding gm1/gm2 ~ 4, which suppresses the voltage noise of Q2 well below that of Q1 and still provides a reduction of input capacitance from ~44 to ~11 pF.

## Noise Measurements

**Figure 4:**
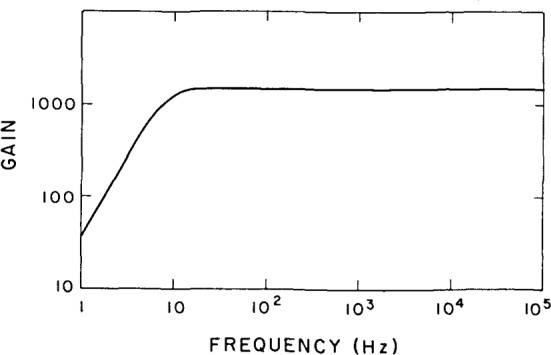
Measured transfer function of the amplifiers.

The amplifier noise was determined by first measuring the transfer function of the amplifier on a spectrum analyzer (see fig. 4). The input capacitance was then obtained by using a known value of the capacitor in series with the input of the amplifier and measuring the change in apparent amplifier gain as a function of capacitance. In order to measure the input current noise, the gate bias, resistor, *R*_g_, was increased to 7×10^11^ Ω so that the term $I_{\text{nQ}1}^{2}Z_{g}^{2}$ would dominate in eq (1). A measurement of the noise from 1.5 to 10 Hz coupled with the known input capacitance, *C*_g_, allows one to write

$$\frac{1}{E_{\text{ni}}\left(f\right)}≃\frac{1}{R_{g}I_{\text{nQ}1}}\left(1+2\pi C_{g}R_{g}f\right),$$ (2)

where *E*_ni_ (*f*) is the equivalent noise at frequency *f* at the input of Q1. Using a linear regression analysis to find the slope, *m*, of the 1*/E*_ni_ vs *f* line, we can then write

$$I_{\text{nQ}1}≃\frac{2\pi C_{g}}{m}.$$ (3)

This analysis holds, assuming that the thermal noise current of *R*_g_ does not swamp the noise current of Q1 and that the perturbation of 1/*f* noise is small. The first assumption is easily checked as our 7×10^11^ Ω resistor used for *R*_g_ generates only $0.15\;\text{fA}/\sqrt{\text{Hz}}$ noise current which is of the same order of magnitude as the noise current associated with Q1. The 1/*f* contribution of current noise in both the input FET and *R*_g_ was measured to be less than $10^{-16}\;A/\sqrt{\text{Hz}}$ at 1.5 Hz.

*E*_nQ1_, the voltage noise associated with Q1, was measured by replacing the 7×10^11^ Ω resistor used for *R*_g_ with a 10 Ω resistor. This makes the $I_{\text{nQ}1}^{2}Z_{g}^{2}$ term in eq (1) insignificant and the equation can then be rewritten to yield:

$$E_{\text{nQ}1}^{2}=E_{\text{ni}}^{2}-E_{nR_{g}}^{2}-{\left(\frac{E_{\text{nQ}2}}{K_{Q1}}\right)}^{2}-{\left(\frac{E_{nR_{d}}}{K_{t}}\right)}^{2}.$$ (4)

If the noise associated with Q2, gm1/gm2, and the output noise are measured, one can infer the noise associated with Q1.

## Results

Measurements using three different 2SK117 FETs for Q1 and a variety of different 2N4116 FETs for Q2 give the following results for the amplifier

$$E_{\text{ni}}≃1.1\;nV/\sqrt{\text{Hz}}\;I_{\text{ni}}≃0.25\;\text{fA}/\sqrt{\text{Hz}}.$$ (5)

**Figure 5:**
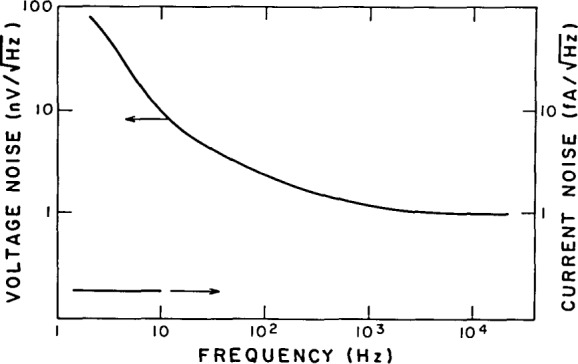
Measured input voltage noise.

Figure 5 shows the measured voltage noise as a function of frequency for the amplifier. Independent measurements with 2N4416 FETs show that the noise voltage associated with them is approximately $3\;\text{nV}/\sqrt{\text{Hz}}$. Using this value and eq (5) we can infer a noise voltage for the 2SK117 of about $0.8\;\text{nV}/\sqrt{\text{Hz}}$. It is interesting to compare this to the theoretical result derived by van der Ziel: [7]

$$e_{\pi Q1}={\left(\frac{2}{3}\frac{4KT}{\text{gm}1}\right)}^{1/2}.$$ (6)

Using $\text{gm}1=\frac{1}{60\;\Omega }$ the transconductance of the 2SK117 at 3 mA drain current, we obtain $e_{\text{nQ}1}=0.82\;\text{nV}/\sqrt{\text{Hz}}$ which is in agreement (possibly fortuitous) with the measured result.

If one measures the gate current of the input FET in a version of this amplifier in which Q2, the common gate portion of the cascode, is shorted, making the input of the amplifier a common source stage, an interesting effect occurs. The gate current, as measured by the voltage drop across *R*_g_, decreases and finally changes sign with increasing drain current. A measurement of the noise current in this region suggests that in fact two (at least) competing currents are responsible, as the noise current is monotonically increasing in the region of apparently zero gate bias current. This is as would be expected for the noise from two competing processes. Thus, this effect is potentially useful in an application in which the amplifier must draw a minimal bias current through the gate. However, a drawback to this circuit is that the input capacitance is ~50 pF as opposed to ~11 pF for the cascode configuration. The cascode amplifier also exhibits very low input bias current, typically less than 0.3 pA for drain currents in the 3 mA range, but it does not exhibit an apparent vanishing of this bias current as does the common source configuration. It should be noted that this effect prevents us from inferring that the noise current in Q1 is due to shot noise in the measured gate current of Q1, since the true gate current is not a well determined quantity in the presence of these competing currents.

The bandwidth of the amplifier as shown in figure 1 is limited to about 500 kHz. This bandwidth limitation is, however, due to the limited bandwidth of the op-amp used for the output stage. If additional bandwidth is required, *R*_c_ and *R*_d_ should be reduced and a video amplifier should be used as the output stage.

## Conclusion

We have discussed the design and test of a FET cascode input amplifier with extremely low voltage noise, less than $1.2\;nV/\sqrt{\text{Hz}}$, and extremely low current noise, $0.25\;\text{fA}/\sqrt{\text{Hz}}$.

This amplifier also has a low input capacitance of 11 pF. Thus it can be used to provide useful low noise gain from 1 Hz to more than 30 MHz. Another significant attribute is the very low bias current drawn by the amplifier, less than 0.3 pA; a modified version of this amplifier draws even less input bias current. A short discussion of design criteria and noise mechanisms in cascode amplifiers is also provided.
